# Mechanisms of carbamate resistance in the malaria vector: *Anopheles gambiae*

**DOI:** 10.1016/j.parepi.2026.e00486

**Published:** 2026-02-19

**Authors:** Wisdom D. Cleanclay, Mosunmola H. Akanni, Tobiloba I. Bajepade, Joshua K. Ajeoge, Suleiman Zakari

**Affiliations:** a Department of Biochemistry, Covenant University, Ota, Ogun State, Nigeria; b Covenant Applied Informatics and Communication - Africa Centre of Excellence, Covenant University, Ota, Ogun State, Nigeria; c Covenant University Public Health and Wellbeing Research Cluster, Covenant University, Ota, Ogun State, Nigeria

**Keywords:** Carbamate, Insecticide resistance, Female *Anopheles gambiae*, Malaria vector

## Abstract

In Africa, the female *Anopheles gambiae* is the primary malaria vector and a key target of vector control measures. The four principal classes of insecticides used in the control of these vectors are pyrethroids, organophosphates, carbamates and organochlorines. Historically, pyrethroids were the main type of insecticides employed to impregnate insecticide-treated nets because they are less toxic to humans and more effective against mosquitoes. The effectiveness of these interventions is however currently challenged by the development of pyrethroid-resistant mosquito populations. The World Health Organization recommends alternative or rotational use of carbamate insecticides in pyrethroid-resistant areas. The mechanism of action of carbamates is to inhibit acetylcholinesterase reversibly to trigger the build-up of acetylcholine in mosquito nerves and consequent paralysis and death of the mosquito. However, carbamate resistance is also on the rise, and poses significant issues to malaria control systems. Notably, the mechanisms of carbamate resistance in *Anopheles gambiae* are target site mutations in the acetylcholinesterase gene and increased detoxification of carbamate by enzymes, including esterases and cytochrome P450s. This review presents a synthesis of existing information on the molecular and metabolic pathways of carbamate resistance in the *Anopheles gambiae* and discuss their consequences for the control of malaria vector. Understanding these resistance mechanisms is crucial for sustaining the effectiveness of IRS, informing insecticide resistance management strategies, and guiding malaria control policies in areas where pyrethroid resistance is increasing. Keywords: Carbamate, Insecticide Resistance, female *Anopheles gambiae*, Malaria Vector.

## 1 Introduction

Malaria is an infectious disease caused by *Plasmodium* parasites, commonly transmitted through the bite of an infected female *Anopheles* mosquito (Sato, 2021). Malaria continues to pose a significant threat, causing high and disturbing rates of sickness and mortality, as regularly reported in the annual publications of the World Malaria Report by WHO; with Africa bearing the highest burden, with an alarming total of 94% of the reported cases (World Malaria Report, 2024). In sub-Saharan Africa, the female *Anopheles gambiae* is recognized as the most efficient malaria transmitting mosquito (Zouré et al., 2021). *Anopheles* mosquito serve as main vectors for human malaria, which affects a significant number of individuals residing in subtropical and tropical countries (Ogunah et al., 2020; Manikandan et al., 2023). The high prevalence of *Anopheles gambiae* particularly during the rainy season correlates with increased malaria transmission and disease burden in affected communities (Oduola et al., 2012).

Vector control interventions, especially the use of insecticide-treated nets (ITNs) and indoor residual spraying (IRS), have been central to malaria prevention efforts. Historically, pyrethroids were the only class of insecticides approved for ITN impregnation due to their efficacy and low toxicity to humans (Zaim et al., 2000). However, the extensive and prolonged use of pyrethroids in both public health and agriculture has led to the widespread emergence of pyrethroid-resistant mosquito populations, significantly undermining the effectiveness of these interventions (Nkemngo et al., 2020). Recent WHO guidelines now recommend the deployment of next-generation nets with dual active ingredients (such as PBO or chlorfenapyr) and advocate for the rotational use of insecticides with different modes of action to manage resistance (World Health Organization, 2021; WHO guidelines for malaria, 2023).

In response to pyrethroid resistance, carbamate insecticides have been introduced, primarily in IRS programs, as alternative or rotational options for vector control (Dengela et al., 2018). Carbamates act by reversibly inhibiting acetylcholinesterase, resulting in the accumulation of acetylcholine, paralysis, and eventual death of the mosquito (Zhao et al., 2014). Although carbamates are not currently used for long-lasting insecticide-treated net (LLIN) impregnation, they remain a critical component of malaria vector control through indoor residual spraying (IRS) and are recommended by the World Health Organization (WHO) as alternative or rotational insecticides in areas experiencing widespread pyrethroid resistance.

With pyrethroid resistance on the rise throughout Africa, IRS increasingly relies on carbamates and organophosphates. Nevertheless, the resistance to carbamates is also observed in the populations of *Anopheles gambiae* in Africa, which is posing a major challenge to the sustainability of malaria control programs (Antonio-Nkondjio et al., 2016).

The health consequences of carbamate resistance to a population are immense. The recent case studies of operations have shown that presence of carbamate resistant *Anopheles gambiae* can cause IRS to fail, resulting in escalation of malaria burden, and outbreaks in areas that were previously controlled (Bamou et al., 2019). West and East Africa have documented resistance that has been linked to decreased efficacy of IRS and this requires urgent changes in the approach of vector control. The resistance mechanisms, including point mutations in the acetylcholinesterase gene (ACE-1) and increased metabolic detoxification are recently properly documented and have a direct impact on the selection and efficiency of insecticides (Binyang et al., 2022; Fagbohun et al., 2020).

Knowledge of molecular and metabolic pathways mediating carbamate resistance is critical to inform policy-makers and maintain the efficacy of IRS-based interventions. This review presents a recent synthesis of existing knowledge on carbamate resistance in *Anopheles gambiae*, which combines novel findings on target-site mutation, metabolic detoxication, and cuticle-mediated resistance to carbamate insecticides. Compared to previous reviews, the study combines resistance mechanisms with reported operational IRS results and compiles regional evidence from several African contexts, which can be used to highlight emerging trends and gaps in the context of malaria control programs.

## 2 Discussion

Carbamate resistance in *Anopheles gambiae* has emerged as a critical public health concern, threatening the continued success of malaria control programs across Africa. The increasing prevalence of resistance to both pyrethroids and carbamates in mosquito populations has profound implications for the effectiveness of vector control interventions, particularly indoor residual spraying (IRS) and insecticide-treated nets (ITNs).

## 3 *Anopheles* vector and parasite biology

**Fig. 1:**
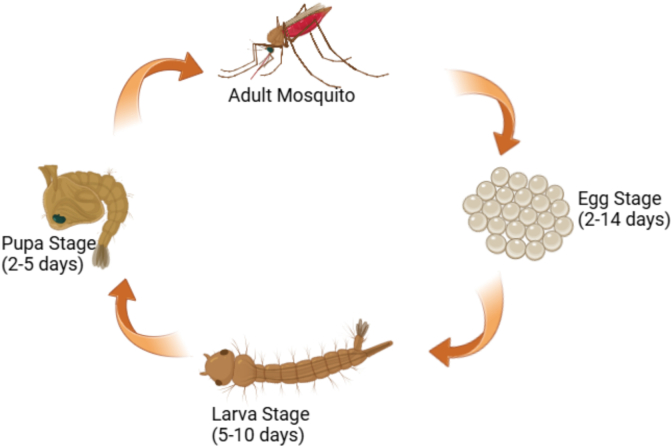
Life cycle of *Anopheles* mosquito.

Mosquitoes represent a major risk to human health because they are responsible for spreading malaria (Vontas et al., 2012). The life cycle of female *Anopheles gambiae* consists of four distinct stages (Fig. 1),which include egg, larva, pupa, and adult (Adhikary et al., 2023). The protozoan parasite *Plasmodium* is the primary cause of malaria (Sato, 2021). In human, malaria is caused by *Plasmodium ovale, Plasmodium vivax, Plasmodium falciparum,* and *Plasmodium malariae* (Ononamadu et al., 2020). These parasites are spread through the blood-feeding habits of adult female *Anopheles* mosquitoes (Adhikary et al., 2023). *Plasmodium* parasites exhibit host specificity, infecting various vertebrates, including mammals, reptiles, humans, and birds. In Humans, *P. falciparum* is the deadliest of the five *Plasmodium* species that lead to malaria and accounts for most malaria-related fatalities, being linked to severe disease complications (Adachi and Tamura, 2020; Bonam et al., 2021). The transmission of *Plasmodium* species between vertebrate hosts typically relies on an insect vector, primarily the female *Anopheles gambiae*. Researchers have identified approximately 200 distinct species of protozoa so far, pathogenic to humans are at least 13 mosquito species among them (Fikadu and Ashenafi, 2023). When infected mosquitoes bite humans, they transmit malaria parasites. These parasites enter the bloodstream and migrate to the liver, where maturation take place. After maturation, they re-enter the bloodstream, invade red blood cells, and multiply. This phase leads to the characteristic symptoms of malaria (Ngotho et al., 2019).

The malaria transmission to mosquitoes and its subsequent spread relies exclusively on the sexual stages of the disease, this understanding is essential to stopping the parasite's spread in Human (Kotepui et al., 2020).

There are more ways that malaria might spread:

(i) Congenital malaria: Congenital malaria occurs when an infected woman transmits parasitized red blood cells to her child either after birth or through the placenta during pregnancy (Tahirou et al., 2020)
(ii) Through blood transfusion: Individuals infected with *P. malariae* can remain contagious for a long time even after recovering from a malaria illness (Sato, 2021).
(iii) Through needle-stick injuries: Incidents of malaria transmission have been reported among drug users or healthcare professionals who share needles. Tragically, some of these cases have resulted in fatal outcomes (Tarantola et al., 2004).

## 4 Preventive strategies for combating malaria

The WHO Global Malaria Programme has introduced the Guidelines for Malaria, which serve as a comprehensive and extensive source of guidance on all matters related to malaria (World Malaria Report, 2022). The WHO Global Technical Strategy for Malaria (2016–2030) establishes a roadmap for malaria elimination and control, aiming to achieve a 90% reduction in global malaria mortality rates by 2030 compared to the 2015 baseline (WHO, 2021). It also aim to serves as a overall guideline on how to direct the action in controlling and eradicating malaria. Originally which was approved by the World Health Assembly in May 2015, and later revised in May 2021, this plan identifies key objectives, milestones, and targets in order to work towards a world free of malaria. In the fight against malaria, the WHO encourages interventions such as control of the vectors, chemoprevention, diagnostic testing, and treatment. These are interventions aim at minimizing the rates of transmission, prevent disease and death related to malaria and, ultimately, the elimination of malaria (WHO, 2021). The global strive to prevent malaria on the basis of Goal 3.3 of the United Nations Sustainable Development Goals (SDGs), complements the WHO Global Technical Strategy for Malaria (Fagbohun et al., 2020).

The transmission and management of malaria are influenced by interactions between humans, parasites, mosquitoes and environment (Savi et al., 2021; Tebamifor et al., 2025; Adegbite et al., 2022). Moreover, past interventions have proven to be unsuccessful in solving the nonlinear dynamics of malaria infection. Failures to explain such dynamics have resulted in unpredictable outcomes and consequently interventions such as the eradication control program, which was abandoned in 1969, and the Global Malaria Control Strategy in 1992, had to be dropped because it failed to produce the desired results (Savi, 2022). The primary malaria control strategy aims at targetting the vectors that carry the disease (Moyes et al., 2020).

Synthetic insecticides are widely used in Africa to combat malaria vectors through methods like indoor residual spraying (IRS) and insecticide-treated mosquito nets (ITNs) (van den Berg et al., 2021). Long-lasting insecticide-treated nets (LLINs) play a primary role in malaria vector control, with indoor residual spraying (IRS) serving as a secondary method (Zouré et al., 2021; Ononamadu et al., 2020). LLINs and IRS are effective in malaria control method by reducing mosquito survival rates and preventing the development of infectious parasites. LLINs continue to be effective even as mosquito tolerance to insecticides increases, as they act as a physical barrier to mosquito feeding (Collins et al., 2019).

Pyrethroids were initially the major insecticide for impregnating bed nets due to pyrethroids low toxicity to animals and high efficacy against insects (Zaim et al., 2000). The widespread use of pyrethroids in public health and agricultural sectors has subjected mosquitoes to strong selective pressure and has led to the manifestation of pyrethroid-resistant mosquitoes (Talipouo et al., 2021). The emerging insecticide resistance observed in *Anopheles* vectors across Africa, undermines the efficiency of insecticide-based vector control methods (Nkemngo et al., 2020).

Carbamates and organophosphates are potential alternatives for mosquito control in cases where pyrethroid resistance is present. To effectively tackle insecticide resistance, the WHO advises rotational use of insecticides with different modes of action or combining different control interventions (WHO guidelines for malaria, 2023).

### 4.1 Carbamate insecticides

**Fig. 2:**
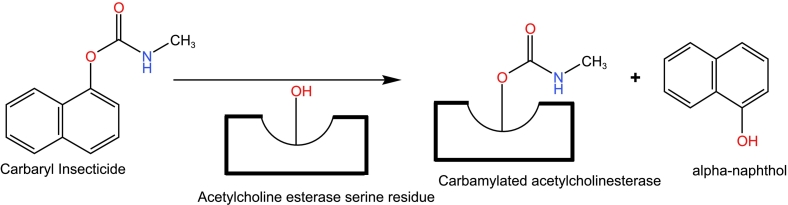
Mechanism of action of Cabaryl Insecticides.

Carbamates are insecticides that competitively inhibit acetylcholinesterase (AChE), by acting as an analogue to acetylcholine and binding to the active site of the enzyme (Zhao et al., 2014). Due to extensive resistance to pyrethroids and organochlorines, carbamates are considered as potential alternatives to combat pyrethroid-DDT resistance. This is primarily because carbamates have a distinct mode of action, inhibiting acetylcholinesterase, which makes them effective against insecticide resistant malaria vectors (Oduola et al., 2012). Managing populations of insecticide-resistant vectors using carbamates remains an important strategy for chemical-based control (Ekedo and Ukpai, 2019). Carbamate insecticides inhibit acetylcholinesterase (AChE) by carbamoylating the serine residue at the active site (Fig. 2), in contrast to phosphorylating it like organophosphates (Barile, 2019; Mdeni et al., 2022). Carbamate insecticides are usually susceptible to hydroxylation due to the formation of reversible bond between AChE active site, and as result, carbamates typically have a relatively short toxicity. Acetylcholinesterase (AChE) enzyme functions by hydrolyzing acetylcholine into choline and acetic acid. This enzymatic action helps to terminate neurotransmitter communication by rapidly breaking down acetylcholine in the synaptic cleft (Reza et al., 2023; Abubakar and Abubakar, 2021). The sustained inhibition of acetylcholinesterase leads to elevated acetylcholine levels, which in turn results in increased neurotransmitter signaling (Abubakar and Abubakar, 2021). Elevated levels of acetylcholine in the central nervous system can lead to various symptoms such as tremor, seizures, hallucinations, confusion, and eventually death of *Anopheles* mosquito (Kaur et al., 2026; Silman, 2021).

Carbamates encompass a diverse range of subtypes, including carbaryl, bendiocarb, propoxur, and many others (Table 1). Each compound within this class has its specific applications, such as insecticide, fungicide, or herbicide, along with distinct characteristics and potential harmful effects (King and Aaron, 2015).

**Table 1:** Examples of Carbamate Insecticides.

NAME OF CARBMATE	STRUCTURE
Propoxur	
Methomyl	
Bendiocarb	
Carbaryl	

### 4.2 Types of carbamates

#### 4.2.1 Propoxur

Propoxur **(Baygon),** also known as 2**-isopropoxyphenyl methylcarbamate** is a carbamate insecticide with the chemical formula C_11_H_15_NO_3_ (Hariprasad and Shetty, 2016; Muda et al., 2018). Propoxur has been approved by the World Health Organization (WHO) for its use against household insects, including mosquitoes. This insecticide functions by inhibiting the action of cholinesterase, which leads to the paralysis of the nervous systems of insects. This mechanism results in a rapid “knockdown” effect, incapacitating the insects quickly (Xie, 2022).

#### 4.2.2 Methomyl

Methomyl **(MET)** is an insecticide belonging to the carbamate-based oxime class employed to control various insect populations (Lin et al., 2020). Methomyl acts by inhibiting the activity of acetylcholinesterase, resulting in the death of insects by causing damage to nerve tissues (Jablonski et al., 2022). The overtime use of methomyl in certain cases has resulted in the increased resistant insect populations (da Silva et al., 2020; Abbas and Shad, 2015; Mutunga et al., 2015; Ibrahim et al., 2016), Methomyl is available in different forms such as soluble concentrates, wettable powders, and water-soluble powders (Phillips et al., 2022). The half-life of methomyl relies on various environmental conditions and can range from days to weeks (Bisht et al., 2015).

#### 4.2.3 Bendiocarb

Bendiocarb, an insecticide belonging to the carbamate family, is extensively employed in public health and agriculture due to its high toxicity. It demonstrates effectiveness to a broad spectrum of disease vector. Bendiocarb is one of the 12 insecticides recommended by the WHO for inclusion in malaria control efforts (Sadasivaiah et al., 2007). Bendiocarb is classified as non-carcinogenic; however, it is highly toxic compared to other carbamates and acts as a reversible inhibitor of acetylcholinesterase. Bendiocarb binds to the acetylcholinesterase active site, causing an accumulation of acetylcholine at nerve-muscle sites, which is crucial for nerve impulse transmission (Ngangue-Siewe et al., 2022).

#### 4.2.4 Carbaryl

Carbaryl, a member of the carbamate family, is primarily utilized as an insecticide. Despite its toxicity to insects, carbaryl is swiftly detoxified and eliminated in vertebrates. It does not accumulate in fat or get secreted in milk, making it a preferred choice for food crops (Fagbohun et al., 2020). Carbaryl exhibits toxicity towards both targeted insects, such as malaria-carrying mosquitos, and beneficial insects like honeybees, as well as crustaceans.

## 5 Mechanism of resistance of female *Anophlese gambiae*

The reliance on carbamates as alternatives to pyrethroids was initially a cornerstone of resistance management strategies (Dossou et al., 2024; Diallo et al., 2022). However, operational evidence now demonstrates that the spread of carbamate resistance is undermining the efficacy of IRS campaigns in several malaria-endemic regions. Studies from West Africa and Cameroon for instance, have shown that high frequencies of the Ace-1 G119S mutation and overexpression of detoxification genes in *An. gambiae* populations are associated with reduced IRS effectiveness (Fagbohun et al., 2020; Elanga-Ndille et al., 2019), increased vector survival and, consequently, a resurgence in malaria transmission. Similar trends have been observed in Nigeria (Fagbohun et al., 2020) and Senegal (Diallo et al., 2022), where resistance has led to operational failures and necessitated rapid changes in insecticide policy.

The efficiency of carbamate interventions is particularly compromised by the development of resistance in malaria vectors (Antonio-Nkondjio et al., 2016). Factors such as the rate, volume of insecticide applications and inherent traits of the mosquito species, also contribute to the development of resistance (Pates and Curtis, 2005; Wood et al., 2010; Abate et al., 2020). Gaining a comprehensive understanding of the underlying resistance mechanisms to carbamate insecticides is essential to protect the limited vector control methods and prevent failure of interventions that rely on carbamates (Diallo et al., 2022). Inadequate targeting of breeding habitats and residential areas with carbamate insecticides allows female *Anopheles gambiae* mosquitoes to grow, develop, and increase in number, emphasizing the need for more comprehensive vector control strategies (Fagbohun et al., 2020).

Carbamate resistance in female *Anopheles gambiae* primarily involves metabolic detoxification of lethal carbamate by enzyme groups such as P450 monooxygenases, esterases and glutathione transferases; decreased target-site sensitivity which slows down the insecticide binding to its target site, particularly acetylcholinesterase (Ace-1) and penetration resistance, which occurs due to the thickening of the mosquito's cuticle (Wood et al., 2010). Another observed mechanism in carbamate resistance is behavioral resistance, whereby mosquitoes avoid contact with insecticides by altering their feeding and resting behaviors. This behavioral adaptation reduces their exposure to carbamate insecticides, thereby limiting the effectiveness of vector control interventions (Meier et al., 2022).

### 5.1 Modification of acetylcholinesterase target site

Target site mutations refer to genetic alteration (Chouaïbou et al., 2017), which involves substitution of glycine for serine at position 119 in the catalytic site of acetylcholinesterase, rendering the enzyme resistant to inhibition by carbamate insecticides (Weill et al., 2003), among malaria vectors in West Africa (Binyang et al., 2022). Previous reports have confirmed the discovery of the acetylcholinesterase (ace-1R) target site mutation G119S in *Anopheles gambiae s.l.* in the South and Centre regions of Cameroon (Binyang et al., 2022; Elanga-Ndille et al., 2019). In 2020, *Anopheles gambiae s.l.* in Gambia were found to be susceptible to bendiocarb, indicating that the mosquitoes had not developed significant resistance to this carbamate (Ononamadu et al., 2020). However, recent studies have detected high frequency of Ace-1 G119S mutation in *Anopheles gambiae* populations across multiple sites in Gambia, indicating widespread resistance to carbamate insecticides (Binyang et al., 2022; Diallo et al., 2022; Tepa et al., 2022). The Ace-1R (G119S) mutation has also been detected in western (Fagbohun et al., 2020) and southern Nigeria, indicating reduced effectiveness of these insecticides in vector control (Oduola et al., 2012).

### 5.2 Metabolic resistance

Enhanced detoxification of insecticides gives rise to metabolic resistance (Oumbouke et al., 2020). Carbamate (bendiocarb) resistance have been linked to the overexpression of multiple P450 genes, which allows mosquitoes to metabolize and detoxify the insecticide (Tepa et al., 2022; Mandeng et al., 2019; Tene-Fossog et al., 2022). Metabolic resistance in mosquitoes arises from increase detoxification of insecticides through over-expression or activity of detoxification enzymes, including glutathione S-transferases (GSTs), cytochrome P450s, and esterases (Ngangue-Siewe et al., 2022; Poupardin et al., 2008). The overexpression of CYP6P3 is observed alongside the detection of phenotypic resistance to bendiocarb (Ngangue-Siewe et al., 2022). Previous research has demonstrated that specific P450 gene, CYP6P3, is capable of metabolizing bendiocarb (Yunta et al., 2019; Adolfi et al., 2019). Enzymes, such as cytochrome P450s, esterases, and GSTs, are upregulated in resistant mosquitoes, allowing them to break down or sequester insecticides thereby reducing their effectiveness (Fagbohun et al., 2020; Simma et al., 2019). Overexpression of these detoxification genes related to insecticide resistance have been detected in field mosquito populations in Côte d'Ivoire (Diouf et al., 2020; Fondjo et al., 2023). This mutation confirms the previous report that two primary mechanisms of insecticide resistance involve target site modification and increased detoxification (Oumbouke et al., 2020) and further emphasizes the need for comprehensive approaches in vector control to address the complex nature of insecticide resistance (Bamou et al., 2019; Mutunga et al., 2015; Ibrahim et al., 2016).

### 5.3 Cuticle resistance

The functional gene involve in the biosynthesis of epicuticular lipids involved in cuticle resistance in insects is CYP4G16. These lipids contributes towards the integrity of the outer cuticular layer, which reduces the penetration of insecticides and increase resistance (Kim et al., 2023). The upregulated expression of the CYP4G16 gene leads to increased levels of cuticular hydrocarbons (CHCs), thereby contributing to insecticide resistance. In resistant female *Anopheles gambiae,* it has been observed that genes associated with reducing insecticide uptake are upregulated (Zouré et al., 2021; Vontas et al., 2020). This flexibility enables them to induce cross-resistance, which adds to multiple insecticide resistance (Mitchell et al., 2012) through limiting the quantity of insecticide that gets into the insect body (Vontas et al., 2020).

#### 5.3.1 Implications for malaria control and need for integrated resistance management

*Reduced IRS Efficacy*: The emergence of carbamate-resistant populations of *An. gambiae* means that post-IRS application mortality rates are lower, and malaria transmission is not curtailed but persists or even increases despite IRS application (Sherrard-Smith et al., 2018).

*Limited Insecticide Options:* With resistance now spanning multiple classes (pyrethroids, carbamates, organophosphates), the arsenal of effective insecticides is shrinking, complicating resistance management and threatening the sustainability of current strategies (Minwuyelet et al., 2025).

*Operational Case Studies:* There have been reports of severe operational failure of IRS in regions such as Côte d'Ivoire (N'Guessan et al., 2003) and Cameroon (Elanga-Ndille et al., 2019), due to carbamate resistance prompting urgent calls for integrated resistance management and the use of next-generation vector control tools.

*Economic and Programmatic Impact:* The necessity to replace cheaper or more readily available insecticides with more expensive ones, the frequency of IRS campaigns, and the threat of malaria outbreaks cause a lot of financial and logistical strain on national malaria control programs.

Given these challenges, strategies to monitor and manage resistance should be put in place at high levels. This includes: (i) Frequent monitoring of resistant marker (e.g. Ace-1 G119S, metabolic gene overexpression) in vector populations (Cleanclay and Opeyemi, 2025). (ii) Rotation of insecticides of alternate modes of action as suggested by the WHO to slow the emergence of resistance. (iii) The introduction of non-chemical measures, including environmental and biological control to lessen dependence on insecticides. (iv) Investments in research and implementation of new instruments, including dual active ingredient nets, and gene-drive technologies to continue to make advances in malaria control.

## 6 Conclusion

The emergence of carbamate-resistant female *Anopheles gambiae* is influenced by the interaction of a complex of mechanisms, such as up-regulation of detoxification genes, the presence of G119S mutation in the Ace-1R, and expression of CYP4G16 resulting in cuticle thickening. All these together compromise the effectiveness of carbamate-based approaches in the control of vectors whether as alternatives or as a combination with pyrethroid insecticides used in Africa. This resistance has posed a major threat to malaria control initiatives and underscores the need to have coordinated and evidence based responses. Based on the evidence in this review, we recommend strengthening resistance monitoring programs and instituting regular surveillance of resistance markers such as Ace-1 mutations and expression of metabolic genes in order to enable timely identification of such resistance and allow for implementation of appropiate intervention. Integrated resistance management must be embraced by the use of rotational or mosaic application of insecticides with different modes of action as suggested by the WHO. These can be complimented by adopting non-chemical measures such as environmental management and reduction of larval source, to reduce dependance on chemical controls/methods. The adoption of policy changes must be informed by data based on operational case studies and surveillance data to ensure that the choice and placement of insecticides meet the local patterns of resistance.

## CRediT authorship contribution statement

**Wisdom D. Cleanclay:** Writing – review & editing, Supervision. **Mosunmola H. Akanni:** Conceptualization. **Tobiloba I. Bajepade:** Writing – review & editing, Data curation. **Joshua K. Ajeoge:** Resources. **Suleiman Zakari:** Writing – review & editing.

## Declaration of competing interest

The authors declare that they have no competing interests.
